# *Rotylenchulus reniformis* Management and Vertical Distribution in Summer-Winter Crop Rotations Including Carinata

**DOI:** 10.2478/jofnem-2025-0052

**Published:** 2025-11-17

**Authors:** Rebeca Sandoval Ruiz, Ramdeo Seepaul, Ian M. Small, Zane J. Grabau

**Affiliations:** Entomology and Nematology Department, Institute of Food and Agricultural Sciences, University of Florida, Gainesville, FL 32611; Laboratory of Nematology, Crop Protection Research Center (CIPROC), Agronomy School, University of Costa Rica, San José 2060, Costa Rica; North Florida Research and Education Center-Quincy, Institute of Food and Agricultural Sciences, University of Florida, Quincy, FL 32351

**Keywords:** *Brassica carinata*, crop rotation, management, reniform nematode

## Abstract

*Brassica carinata* (carinata) is an emerging winter biofuel crop in the southeastern United States. *Rotylenchulus reniformis* (reniform nematode) is an important yield-robbing parasite on cotton in the region. A better understanding of rotation systems involving carinata would guide *R. reniformis* management and crop selection decisions. This study aimed to determine the effect of winter crop rotations with or without carinata, in combination with summer crops, on *R. reniformis* at two soil depths in a field study in North Florida. Two-year winter rotations included fallow-carinata, fallow-fallow, and oat-carinata. Winter rotations were crossed with corn, cotton, soybean, and peanut each year. Soil samples were taken from 0–30 cm and 30–120 cm depth after both summer and winter crop harvest for 4 yr. *Rotylenchulus reniformis* soil abundances were greater at 0–30 cm than at 30–120 cm deep, but crop effects were generally similar at both depths. Cotton supported greater *R. reniformis* soil abundances than corn, peanut, or soybean. Winter rotations did not consistently affect *R. reniformis*, regardless of prior summer crop, although carinata tended to decrease *R. reniformis* soil abundances. In summary, carinata production expands options for winter crop rotations to manage *R. reniformis*.

*Rotylenchulus reniformis* Linford and Oliveira, 1940 (reniform nematode, *R. reniformis*) is a major cotton (*Gossypium hirsutum* L.) parasite (Lawrence, 2021). The economic impact of *R. reniformis* on cotton yield has been estimated at >US$100 million, with the potential to reduce yield by 50% in individual fields with severe infestation (Dyer et al., 2020). *R. reniformis* is a sedentary semi-endoparasite that feeds on roots (Robinson et al., 1997), impairing water and nutrient uptake, triggering morphological and physiological dysfunction, and increasing susceptibility to other plant diseases (Robinson, 2007; Crow et al., 2020). Because *R. reniformis* can persist deep in the soil profile, conventional management strategies that treat only the topsoil may have reduced efficacy (Robinson et al., 2005).

While there are existing strategies for *R. reniformis* management, additional options are still needed. Along with nematicide application (Gowen, 1997; Oka et al., 2009; Ntalli and Caboni, 2017) and the emerging use of resistant cultivars (Khanal et al., 2018; Singh et al., 2022), crop rotation with poor or non-hosts are a pivotal component of an integrated nematode management program for *R. reniformis*. Cash crops such as corn (*Zea mays* L.), peanut (*Arachis hypogaea* L.), resistant soybean (*Glycine max* L.), and wheat (*Triticum aestivum* L.) (Westphal and Scott, 2005; Robinson, 2007; Stetina et al., 2007), as well as forage or cover crops such as oat (*Avena sativa* L.), sorghum (*Sorghum bicolor* L.), and bahiagrass (*Paspalum notatum* Flüggé), may help with *R. reniformis* management (Robinson, 2007; Schumacher et al., 2020, 2024; Singh et al., 2023).

However, the availability of agronomically and economically viable rotation crops is one limitation for identifying effective crop rotation practices to manage *R. reniformis*; therefore, emerging crops are particularly relevant for this purpose. *Brassica carinata* A. Braun (carinata), a non-food biofuel crop, is one such emerging winter crop in the southeastern United States that could help diversify crop rotation options. Carinata is well-suited to production in the Southeast as it is tolerant to extreme environmental conditions (Klíma et al., 2012; Chang et al., 2015; Majidi et al., 2015) and some pathogens or pests (Getinet et al., 1996; Tonguc and Griffiths, 2004; Subramanian et al., 2005). It could fit into current southeastern row cropping systems, offering the possibility to profitably farm >1.4 million ha that are typically fallow in winter (Seepaul et al., 2021; Iboyi et al., 2023). Although carinata is an emerging biofuel crop, research on its interactions with plant-parasitic nematodes – particularly *R. reniformis* – remains limited.

In terms of nematode management, carinata is part of the Brassicaceae family (Warwick, 2011), known for having a glucosinolate-myrosinase defense system called “mustard oil bomb” (Angelino et al., 2015), which can release compounds toxic to some soil pests, including nematodes (Waisen et al., 2020). Carinata has a robust (Barro and Martin, 1999) and deep root system, reaching up to 90 cm deep (Lal et al., 2019), although most roots are in the top 30 cm of soil (Seepaul et al., 2019), which may be beneficial for managing *R. reniformis* deeper in the soil profile. Recent greenhouse studies showed that carinata is a poor *R. reniformis* host (Sandoval-Ruiz and Grabau, 2023a), and its dry residue applied at 2% w-w could help manage *R. reniformis* populations (Sandoval-Ruiz and Grabau, 2023b). However, in another greenhouse study, carinata was worse than the poor host oat for managing *R. reniformis* following a combination of rotation and incorporation of dry or fresh organic matter (Sandoval-Ruiz and Grabau, 2023c). Due to these mixed results and because crop-nematode dynamics may vary somewhat from greenhouse to field conditions, field evaluation of carinata for *R. reniformis* management is needed.

In the southeastern United States, winter crops such as carinata are not grown in isolation; they are always part of a larger rotation with summer crops (e.g., cotton, corn, peanut, soybean). *R. reniformis* persists across seasons and its population dynamics depend on the previous crop's host status (Shumacher et al., 2024). Therefore, although the host status of these summer crops for *R. reniformis* is already known (Westphal and Scott, 2005; Robinson, 2007; Stetina et al., 2007), evaluating winter rotations in combination with common summer crops – rather than after a single summer crop – provides a more complete picture of how cropping systems manage *R. reniformis*. For interpreting any winter crop-summer crop interactions, it is important to verify *R. reniformis* population trends in summer crops in this study, even if host status for a summer crop has been previously reported. In addition, *R. reniformis* population dynamics for these summer crops at varying depths have not been examined despite the known importance of *R. reniformis* residing deep in the soil profile (Robinson et al., 2005).

This study investigated both the vertical distribution of *R. reniformis* populations and the management outcomes of including carinata in crop rotations. Specifically, the objectives were to determine the effects of winter crop rotations – including those with carinata – and common summer crops on *R. reniformis* soil abundances at various depths in the soil profile.

## Materials and Methods

### Location

This research was conducted at the University of Florida North Florida Research and Education Center (30°32′29.41″ N, −84°35′12.30″ W) outside of Quincy, FL, on a loamy sand soil (86.5% sand, 10.4% clay, 3.04% silt) from a Dothan-Fuquay complex. This site had an endemic infestation of *R. reniformis*.

### Experimental design

This study used a randomized complete block design with four replications in a split-split plot arrangement, with winter rotation as the main plot factor, summer crop as the subplot factor, and depth as the sub-subplot factor. Each subplot was 12.2 m long by 11 m wide. There were 2.4-m fallow alleys between each plot in the same replicate and 9-m alleys between each replicate. The population densities of *R. reniformis* at 0–30 cm and 30–120 cm deep were determined from soil core sampling as described later. The winter crops carinata, oat, and a bare fallow were rotated in 2-yr cycles of carinata-fallow (Ca-F), fallow-fallow (F-F), and oat-carinata (O-Ca). Two winter rotation cycles (4 yrs) were completed, and winter rotation treatments were not re-randomized between cycles, so the same rotations were maintained in the same plots throughout the study (Table 1). Winter rotations were crossed with corn, soybean, cotton, and peanut summer crops, which were rotated in that order in a 4-yr rotation. This summer rotation constituted four summer crop treatments, each beginning with a different crop, such that each summer crop was present each year. The rotation was initiated in Winter 2016–17, but samples were taken starting at the end of Summer 2017. Cropping sequences are described in Table 1.

**Table 1:** Winter-summer cropping sequence for crop rotation field trial conducted near Quincy, FL.

**Rotation^a^**	**Year 1 (2017–18)**		**Year 2 (2018–19)**		**Year 3 (2019–20)**		**Year 4 (2020–21)**	

	**Summer 1**	**Winter 1**	**Summer 2**	**Winter 2**	**Summer 3**	**Winter 3**	**Summer 4**	**Winter 4**
1	Corn	Fallow	Soybean	Fallow	Cotton	Fallow	Peanut	Fallow
2	Soybean		Cotton		Peanut		Corn	
3	Peanut		Corn		Soybean		Cotton	
4	Cotton		Peanut		Corn		Soybean	

5	Corn	Fallow	Soybean	Carinata	Cotton	Fallow	Peanut	Carinata
6	Soybean		Cotton		Peanut		Corn	
7	Peanut		Corn		Soybean		Cotton	
8	Cotton		Peanut		Corn		Soybean	

9	Corn	Carinata	Soybean	Oat	Cotton	Carinata	Peanut	Oat
10	Soybean		Cotton		Peanut		Corn	
11	Peanut		Corn		Soybean		Cotton	
12	Cotton		Peanut		Corn		Soybean	

a Individual rotation treatments (rows) were on the same replicated plots during the entire study.

### Crop production

The crop cultivars used in the experiments were: ‘Avanza 641’ carinata, ‘Coker 227’ oat, ‘Pioneer 1197YHR’ corn from year 1 to year 3, and ‘Pioneer 1870YHR’ in year 4, ‘DP1646 B2XF’ cotton, and ‘Georgia 06G’ peanut. Soybean varieties were ‘Pioneer P55T81R’ during year 1, ‘Pioneer P52A26R’ in years 2 and 3, and ‘Pioneer P76T54R2’ in year 4. Soybean and corn cultivars were changed during the trial due to seed availability and in an effort to improve soybean productivity. Oat and carinata were planted mechanically with 30.5 cm row spacing at 6.72 kg/ha. Summer crops were planted using 91 cm row spacing. Within rows, summer crops were seeded at 13 cotton seeds/m, 8 corn seeds/m, and 20 peanut or soybean seeds/m. The trial was irrigated as needed by a traveling overhead sprinkler irrigation gun. Pest and soil fertility management practices varied by crop and were based on common practices for those crops in the Southeast (Wright et al., 2021, 2022a, 2022b, 2022c). Each year, terbufos (1.21 kg/ha) nematicide-insecticide was applied in-furrow for corn. Similarly, phorate insecticide was applied in-furrow for cotton and peanut at 1.21 kg/ha and 1.12 kg/ha, respectively, each year. Soybean received in-furrow insecticide (chlorpyrifos at 1 kg/ha) only in year 4. These granular pesticides were delivered onto the seed in the open planting furrow through tubes. Oat was not harvested but rather terminated with glyphosate at 2.34 L/ha. Details about planting and harvesting dates for each crop are provided in Table 2.

**Table 2:** Planting and harvest dates for summer and winter rotation combinations each year in a field trial near Quincy, FL.

**Season**	**Crop**	**Planting 1**	**Planting 2**	**Harvest 1**	**Harvest 2**	**Soil sampling for nematodes**
Summer 1 (2017)^a^	Cotton	28 April 17	14 June 17	27 September 17	29 November 17	4–5 December 17
	Peanut	08 May 17	12 June 17	27 September 17	01 November 17	
	Soybean	22 May 17	14 June 17	20 November 17	20 November 17	
	Corn	05 April 17	12 June 17	16 August 17	29 October 17	

Winter 1 (2017–18)	Carinata	13 December 17		04 June 18		5–6 June 18
	Oat					

Summer 2 (2018)^a^	Corn	05 April 18	12 June 18	16 August 18	29 October 18	19–20 November 18
	Cotton	27 April 18	06 June 18	08 October 18	31 October 18	
	Peanut	04 May 18	12 June 18	04 October 18	29 October 18	
	Soybean	24 May 18	11 June 18	19 November 18	19 November 18	

Winter 2 (2018–19)	Carinata	08 January 19		29 May 19		3–4 June 19
	Oat	13 December 18		12 March 19		

Summer 3 (2019)^a^	Corn	18 March 19	10 June 19	05 August 19	27 September 19	14–15 November 19
	Cotton	29 April 19	10 June 19	27 September 19	29 May 19	
	Peanut	09 May 19	10 June 19	01 October 19	25 October 19	
	Soybean	17 May 19	10 June 19	15 November 19	15 November 19	

Winter 3 (2019–20)	Carinata	16 December 19		19 May 20		27–28 May 20
	Oat					

Summer 4 (2020)^a^	Corn	13 March 20	22 May 20	15 July 20	9 September 20	13 November 20
	Cotton	22 March 20	22 May 20	08 October 20	5 November 20	
	Peanut	05 May 20	22 May 20	06 October 20	26 October 20	
	Soybean	11 May 20	22 May 20	9 November 20	9 November 20	

Winter 4 (2020–21)	Carinata	17 November 20		17 May 21		20 May 21
	Oat	24 November 20		12 March 21		

a Planting 1 and harvest 1 correspond to the crop planting/harvest after the winter rotation with fallow or oat. Planting 2 and harvest 2 correspond to the crop planting/harvest after carinata.

### Soil sampling for nematodes

Soil samples for nematode analysis were collected twice each annual summer-winter cropping cycle: (i) after summer crop harvest, and (ii) after winter crop harvest (Table 1). Sampling was conducted for four growing cycles (Years 1 to 4) from 2017 to 2021. Sampling dates are abbreviated as a combination of the completed cropping season and year of rotation (e.g., Winter 1) as summarized in Tables 1 and 2.

The field conditions, including rainfall and irrigation as well as air and soil temperature, are indicated in Table 3. At each sampling date, two soil cores (120 cm depth × 4.5 cm diameter) from each subplot were collected in a polyvinyl chloride (PVC) liner using a truck-mounted hydraulic probe (Geoprobe, Geoprobe Systems, Salina, KS). Cores were taken near root systems. Subsequently, the PVC liners were cut lengthwise, and the soil from each plot was separated by depth (from 0–30 cm to 30–120 cm). From each subplot, the two soil cores from a given depth were pooled and screened by hand using a soil sifter with a 0.41 cm^2^ size wire mesh to homogenize before nematode extraction.

**Table 3:** Monthly environmental conditions at the field trial site during the study.^a^

	**Month**	**Jan^b^**	**Feb**	**Mar**	**Apr**	**May**	**Jun**	**Jul**	**Aug**	**Sep**	**Oct**	**Nov**	**Dec**
2017	Rainfall + irrigation (cm)				0.3	0.5	0.8	3.3	3.3	0.3	1.7	0.0	0.3
	Air T (°C)				20.4	22.8	24.6	26.3	26.3	24.2	20.8	15.5	12.2
	Soil T (°C)				21.6	24.3	25.3	26.8	26.7	24.7	22.4	17.1	12.9

2018	Rainfall + irrigation (cm)	0.2	0.5	0.4	0.2	3.2	3.7	3.7	2.2	2.2	0.4	0.7	0.9
	Air T (°C)	8.2	17.6	14.7	18.1	23.7	26.1	26.2	25.7	26.1	22.1	14.2	12.8
	Soil T (°C)	8.9	15.9	15.4	18.9	24.6	26.4	26.7	26.1	26.4	22.3	14.7	12.2

2019	Rainfall + irrigation (cm)	1.0	0.1	1.7	0.5	4.6	0.4	2.0	2.2	6.8	0.4	0.1	0.4
	Air T (°C)	11.2	16.5	15.3	19.0	24.6	26.2	26.1	26.4	26.4	22.5	13.4	13.4
	Soil T (°C)	10.9	14.9	16.0	19.4	26.1	27.2	27.2	27.1	27.2	23.1	14.2	12.8

2020	Rainfall + irrigation (cm)	0.2	0.5	1.7	0.4	4.9	1.8	4.5	1.9	1.3	0.2	0.3	0.3
	Air T (°C)	13.0	14.2	20.3	20.0	22.7	25.8	26.7	26.7	24.6	22.1	18.1	10.3
	Soil T (°C)	12.5	13.1	18.8	20.7	23.7	25.6	27.0	27.4	23.8	21.2	17.2	9.7

2021	Rainfall + irrigation (cm)	0.6	0.6	0.3									
	Air T (°C)	11.4	13.1	14.0									
	Soil T (°C)	10.4	12.2	14.2									

a Data are provided from the FAWN weather station at University of Florida North Florida Research and Education Center near Quincy, FL. https://fawn.ifas.ufl.edu/.

b Rainfall and irrigation are total per month. Soil and air temperatures are monthly averages.

FAWN, Florida Automated Weather Network.

### Nematode extraction and identification

Nematodes were extracted from 100 cm^3^ soil using the sucrose centrifugal floatation method (Jenkins, 1964). Samples were fixed in 2% formalin and then counted and identified using a 400× inverted microscope (Primovert, Carl Zeiss Inc., Thornwood, NY). The total nematode soil abundance was recorded; the first 200 nematodes were identified morphologically (Mai and Mullin, 1996); and absolute nematode abundance per 100 cm^3^ soil was calculated as in Schumacher et al. (2020). Based on study objectives, only *R. reniformis* abundances are reported here. In addition to *R. reniformis*, *Nanidorus* spp. was common, and *Helicotylenchus*, *Meloidogyne*, *Mesocriconema*, *Pratylenchus*, and *Xiphinema* were detected in low abundances.

## Data analysis

Statistical analysis was done with RStudio version 2021.09.0 (The R Foundation for Statistical Computing, Vienna, Austria). Data were analyzed separately for each sampling date using a three-way ANOVA with a split-split plot arrangement (McIntosh, 1983) with winter rotation as the main plot, summer crop as the subplot, and depth as the sub-subplot factors, respectively. Replicates were considered random effects in the ANOVA model. Replicate × winter rotation was the error term for winter rotation, replicate × winter rotation × summer crop was the error term for summer crop, and winter rotation × summer crop, and residual error was the error term for depth, depth × winter rotation, and depth × summer (McIntosh, 1983). Assumptions for the ANOVA models were checked using Levene's Test for homogeneity, and normal probability plots for normality of the residuals (Levene, 1960; Cook and Weisberg, 1999). Nematode abundances were transformed by ln(x + 1) to meet the normality assumption. For variables with significant (*P* ≤ 0.1) main effects (winter rotation, summer crop, or depth), mean separation was done by Fisher's protected LSD (α = 0.05). Significant (*P* ≤ 0.1) two-way interactions (winter rotation by depth, summer crop by depth, or summer crop by winter rotation) were assessed using a split-plot analysis as described for the main effects. When there was significant winter rotation by depth or summer crop by depth interactions, both the main effects of depth within each crop and main effects of crop within each depth were analyzed. For summer by winter crop interactions, the main effects of winter crops were analyzed within each summer crop. When there were 3-way interactions (depth × winter rotation × summer crop), the main effects of winter crop were analyzed individually within each depth-summer combination using one-way ANOVA. Untransformed means are presented in the result section and figures.

## Results

### Depth effects on *R. reniformis*

**Figure 1:**
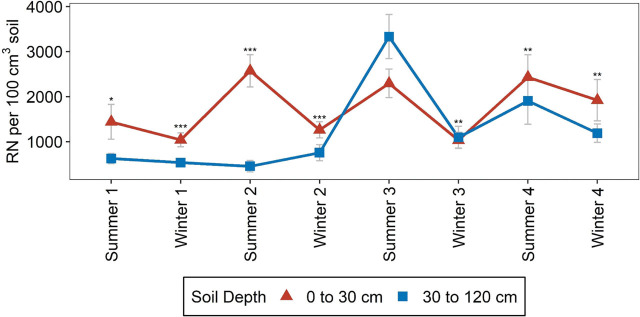
*Rotylenchulus reniformis* (RN) soil abundance by season at 0–30 cm and 30–120 cm, as affected by depth. Mean values and standard errors are presented. Asterisks above the mean denote significant differences between soil depths, within each season, based on ANOVA, *P*-value ≤0.01 (^***^), *P*-value ≤0.05 (^**^), *P*-value ≤0.1 (^*^). “Summer” and “Winter” indicate soil samples collected at harvest of summer and winter crops, respectively. The number following a season indicates the year during study when sampling took place. Error bars represent standard errors.

**Figure 2:**
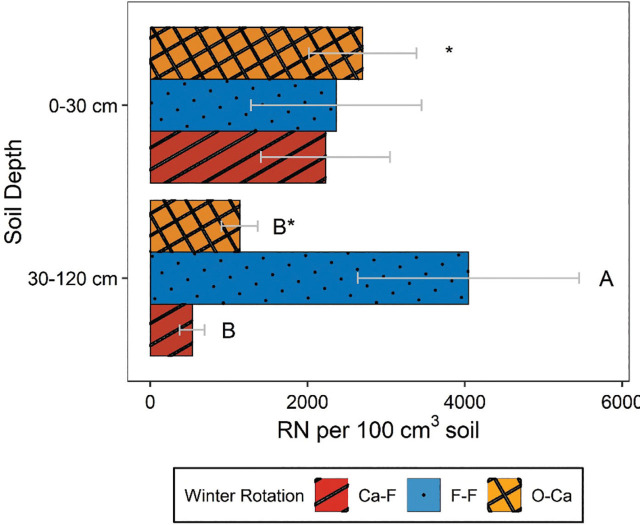
Interactive effects of soil depth and winter crop rotation on *Rotylenchulus reniformis* (RN) soil abundances from sampling around harvest of summer crops in Year 4 of field study. “Ca-F,” “F-F,” and “O-Ca” are 2-yr winter rotations of carinata-fallow, fallow-fallow, and oats-carinata, respectively. Letters next to means denote significant differences among treatments, within a depth in the soil profile, based on Fisher's protected LSD, *P*-value ≤0.05. ^*^Indicates significant depth effects (ANOVA, *P* < 0.05) within the given winter rotation. Error bars represent standard errors.

*Rotylenchulus reniformis* abundances were significantly greater in the top 30 cm of the soil than in the 30–120 cm section in every season except Summer 3 (Table 4, Fig. 1). In Summer 3 and Summer 4, there were significant depth by winter rotation interactions (Table 4), but depth effects did not vary significantly within any individual winter rotation in Summer 3 (data not shown). In Summer 4, depth effects did vary by winter rotation, with greater *R. reniformis* abundances at 0–30 cm than 30–120 cm depth only in the Ca-F rotation (Fig. 2).

**Table 4:** Influence of winter rotation, summer crop, and depth in soil profile on *Rotylenchulus reniformis* soil abundances based on *P*-values from ANOVA.

**Main factors**	**Summer 1**	**Winter 1**	**Summer 2**	**Winter 2**	**Summer 3**	**Winter 3**	**Summer 4**	**Winter 4**
Winter rotation (W)	0.42	0.09^*^	0.33	0.11	0.52	0.93	1.96E−03^***^	0.63
Summer crop (S)	0.04^**^	2.73E−03^***^	4.19E−06^***^	6.3E−05^***^	0.01^**^	4.36E−03^***^	0.06^*^	0.03^**^
Depth (D)	0.10^*^	1.96E−03^***^	5.73E−11^***^	3.32−05^***^	0.73	0.02^**^	1.88E−08^***^	0.06^*^

Interactions								

W × S	0.96	0.88	0.02^**^	0.08^*^	0.7	0.58	0.83	0.42
W × D	0.66	0.86	0.17	0.25	0.08^*^	0.25	2.47E−04^***^	0.71
S × D	0.19	0.78	0.74	0.2	2.08E−03^***^	0.05^**^	0.01^***^	0.06^*^
W × S × D	0.55	0.84	0.3	0.67	1	0.7	0.11	0.42

*** *P*-value ≤0.01,

** *P*-value ≤0.05,

* *P*-value ≤0.1, and blank = *P*-value >0.1.

Depth effects varied by summer crop in Summer 3, Winter 3, Summer 4, and Winter 4 (Table 4). In Winter 4, there were no significant depth effects within any individual summer crop (Table 5). For Summer 3, Winter 3, and Summer 4, depth effects did vary by summer crop (Table 5), with *R. reniformis* more abundant deeper in the soil profile for the peanut phase in Summer 3 and Winter 4, which contrasts the overall trend. In Summer 4, *R. reniformis* abundance was greater in the shallow soil profile for corn and soybean only (Table 5).

**Table 5:** Effect of summer crop on *Rotylenchulus reniformis* (RN) soil abundances (nematodes/100 cm^3^ soil) at different depths in the soil profile across seasons.

**Summer crop**	**Depth (cm)**	**RN/100 cm^3^ soil^a^**	**Summer crop**	**Depth (cm)**	**RN/100 cm^3^ soil**
**Summer 3**					

Corn	0–30	1,771 b	Corn	30–120	2,045 B
Cotton	0–30	3,944 a	Cotton	30–120	4,033 A
Peanut	0–30	1,277 b^*^	Peanut	30–120	4,963 A^*^
Soybean	0–30	2,180 ab	Soybean	30–120	2,283 B

**Winter 3**					

Corn	0–30	672 b	Corn	30–120	506 B
Cotton	0–30	2,152 a	Cotton	30–120	2,153 A
Peanut	0–30	638 b^*^	Peanut	30–120	1,256 A^*^
Soybean	0–30	669 ab	Soybean	30–120	461 B

**Summer 4**					

Corn	0–30	776 c^*^	Corn	30–120	643 B^*^
Cotton	0–30	6,005 a	Cotton	30–120	4,624 A
Peanut	0–30	783 c	Peanut	30–120	1,315 AB
Soybean	0–30	2,159 b^*^	Soybean	30–120	1,038 AB^*^

**Winter 4**					

Corn	0–30	956 b	Corn	30–120	617 B
Cotton	0–30	4,233 a	Cotton	30–120	1,345 A
Peanut	0–30	1,003 b	Peanut	30–120	1,543 A
Soybean	0–30	1,485 ab	Soybean	30–120	1,238 A

a Lowercase and uppercase letters indicate significant differences among summer crops (Fisher's protected LSD, α = 0.05) within each depth and season.

* Indicates significant differences between depths within a given crop and season (ANOVA, *P* < 0.05).

### Winter rotation effects on *R. reniformis*

**Figure 3:**
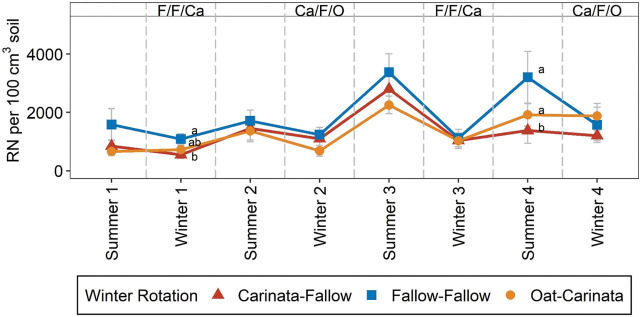
*Rotylenchulus reniformis* (RN) soil abundances by season, as affected by winter rotation. Mean values and standard errors are presented. Letters next to means denote significant differences among treatments, within each season, based on Fisher's protected LSD, *P*-value ≤0.05. Letters at the top of the graph (Ca: carinata, F: fallow, O: oat) represent the winter crop present for the corresponding winter season, for Ca-F, F-F, and O-Ca rotations, respectively. “Summer” and “Winter” indicate soil samples collected at harvest of summer and winter crops, respectively. The number following a season indicates the year during study when sampling took place. Error bars represent standard errors.

*Rotylenchulus reniformis* abundances were not consistently affected by winter rotations (Table 4, Fig. 3). *R. reniformis* abundances tended to be numerically greater in the F-F rotation compared to O-Ca or Ca-F. Across seasons, the Ca-F treatment resulted in numerical reductions in *R. reniformis* populations ranging from 8% to 57% relative to F-F, while the O-Ca treatment showed reductions ranging from 8% to 45%, except that it numerically increased *R. reniformis* abundances 19% in Spring 2021 (Fig. 3). However, the only significant differences in winter rotation were in Winter 1 and Summer 4. In Winter 1, F-F had significantly greater *R. reniformis* soil abundances than Ca-F, with O-Ca intermediate. In Summer 4, F-F and O-Ca had greater *R. reniformis* abundance than Ca-F (Fig. 3). In Summer 2 and Winter 2, the influence of winter rotation varied by summer crop, with *R. reniformis* abundances less for O-Ca than Ca-F or F-F only in combination with corn (Table 6). In Summer 4, winter rotation effects varied by soil depth, with *R. reniformis* soil abundances greater for F-F than O-Ca and Ca-F only at the 30–120 cm soil depth (Fig. 2).

**Table 6:** Effects of winter rotation and summer crop on *Rotylenchulus reniformis* (RN) soil abundances across seasons.

**Summer crop**	**Winter rotation^a^**	**RN/100 cm^3^ soil^b^**	**Winter rotation**	**RN/100 cm^3^ soil**	**Winter rotation**	**RN/100 cm^3^ soil**
	**Summer 2**					
Corn	Ca-F	1,181 b Y	F-F	1,956 Y	O-Ca	43 c Z
Cotton	Ca-F	3,292 a	F-F	2,153	O-Ca	3,841 a
Peanut	Ca-F	900 b	F-F	1,294	O-Ca	791 b
Soybean	Ca-F	450 b	F-F	1,456	O-Ca	793 b
	
	**Winter 2**					
	
Corn	Ca-F	550 b Y	F-F	1,277 Y	O-Ca	155 b Z
Cotton	Ca-F	2,791 a	F-F	2,250	O-Ca	1,561 a
Peanut	Ca-F	487 b	F-F	610	O-Ca	600 a
Soybean	Ca-F	563 b	F-F	819	O-Ca	420 a

a “Ca-F”, “F-F”, and “O-Ca” are 2-yr winter rotations of carinata-fallow, fallow-fallow, and oats-carinata, respectively.

b Lowercase letters indicate significant differences among summer crops (Fisher's protected LSD, α = 0.05) within a given season and winter rotation. Uppercase letters indicate significant differences among winter rotations within a given summer crop and season. Means were separated by Fisher's protected LSD (α = 0.05).

### Summer crop effects on *R. reniformis*

**Figure 4:**
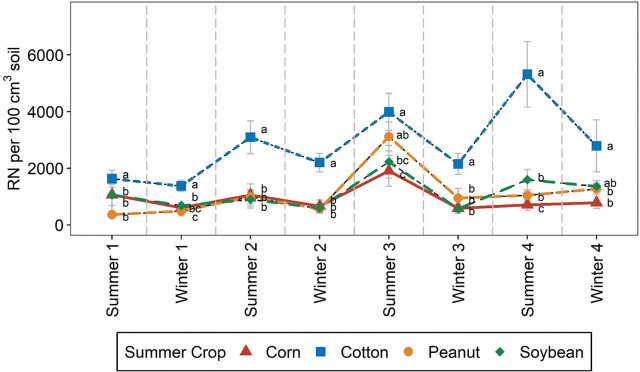
*Rotylenchulus reniformis* (RN) soil abundances by season, as affected by summer crop. Mean values and standard errors are presented. Letters next to means denote significant differences among treatments, within each season, based on Fisher's protected LSD, *P*-value ≤0.05. “Summer” and “Winter” indicate soil samples collected at harvest of summer and winter crops, respectively. The number following a season indicates the year during study when sampling took place. Error bars represent standard errors.

Summer crop significantly affected *R. reniformis* each season with *R. reniformis* soil abundances significantly greater for cotton than all other summer crops in most seasons (Table 4, Fig. 4). The only exceptions were Summer 3 when *R. reniformis* abundances were significantly greater for cotton than soybean or corn only and Winter 4 when *R. reniformis* abundances were significantly greater for cotton than corn and peanut only (Fig. 4). There were no significant differences in *R. reniformis* abundances among corn, peanut, and soybean in most seasons (Fig. 4).

Summer crop effects varied between soil depths in Summer 3 and 4 and Winter 3 and 4 (Tables 4 and 5). Across those seasons, in the shallow soil profile, cotton consistently had greater *R. reniformis* abundances than peanut or corn, with soybean statistically similar to cotton except in Summer 4 (Table 5). In contrast, in the deeper soil profile, cotton and peanut generally had greater *R. reniformis* abundances than other crops in seasons with a summer crop by depth interaction (Table 5).

Summer crop effects varied by winter rotation in Summer 2 and Winter 2, with significant summer crop effects only in combination with Ca-F and O-Ca winter rotations (Tables 4 and 6). In Summer 2, *R. reniformis* abundances were greater for cotton than any other summer crop for Ca-F and O-Ca only (Table 6). In Winter 2, *R. reniformis* abundances were greater for cotton than any other summer crop for Ca-F, but less for corn than other summer crops for O-Ca (Table 6).

## Discussion

*Rotylenchulus reniformis* was consistently more abundant shallow in the soil profile (0–30 cm) compared to deeper in the soil profile (30–120 cm depth). This is consistent with previous reports of higher *R. reniformis* abundances in the top 30 cm of the soil (Holguin et al., 2015; Schumacher et al., 2024). Despite being less abundant in the deeper layer, *R. reniformis* was still present below the plow layer (top 30 cm of the soil profile), which is documented in other studies (Westphal et al., 2004; Robinson et al., 2005) and is below the conventional nematode sampling depth. Crop rotation can help, or harm, *R. reniformis* management to at least 120 cm deep in the soil profile, as rotation effects were generally similar across soil depths.

Winter rotations were inconsistent in managing *R. reniformis*, with fallow tending to increase *R. reniformis*, but not consistently. *R. reniformis* feeding on weeds in the fallow treatment is the likely explanation for intermittently increased *R. reniformis* abundances in that rotation. This has been observed with plant-parasitic nematodes in other studies, such as *Meloidogyne javanica* increasing in fallow relative to other crops in rotation before ginger (Stirling et al., 2012). The fact that both winter crops in the study, oat and carinata, are poor host crops for *R. reniformis* (Robinson, 2007; Sandoval-Ruiz and Grabau, 2023a), contributed to lack of differences among winter rotations, but also reflect grower practices as oat and other small grains are common winter cover crops in the area. Environmental conditions during the trial were generally within typical ranges, particularly in the winter season when carinata was grown (Table 3). Mild and consistent air and soil temperatures, along with adequate rainfall and irrigation inputs, suggest that *R. reniformis* responses were mostly due to biological factors such as host status and weed presence and not washed out by extreme weather.

From a practical perspective, this study should increase confidence for including carinata in rotations on *R. reniformis*-infested land as it is at least similar to current winter rotation options for *R. reniformis* management. While improvement over current options would be preferred, carinata diversifies options for managing *R. reniformis*, which has value. While carinata did not consistently manage *R. reniformis* better than fallow, growing carinata does provide additional benefits for soil and water conservation (Adetunji et al., 2020), as well as the potential for increased income (Iboyi et al., 2023), compared to leaving the soil fallow.

Efficacy at *R. reniformis* management varied by summer crop. Cotton generally supported greater *R. reniformis* soil abundances than corn and peanut. This is consistent with prior host status (Robinson, 2007) and crop rotation field research (Stetina et al., 2007; Schumacher et al., 2024). The terbufos nematicide that was applied in corn could have enhanced management of *R. reniformis* as there are mixed reports of this pesticide decreasing populations of this nematode (Lawrence et al., 1990; Silva et al., 2025). However, *R. reniformis* management by corn should be attributed primarily to its known status as a poor host for this nematode (Stetina et al., 2007).

Most soybean cultivars are good hosts for *R. reniformis* (Robinson et al., 1997), so it was unexpected that soybean often supported similar *R. reniformis* abundances to poor hosts (peanut and corn). However, soybean cultivars can vary in their susceptibility to *R. reniformis* (Robbins et al., 1994), and the cultivars used in this trial could be less susceptible than other cultivars as their host status for *R. reniformis* is unknown. Hence, growers using a soybean phase in rotation should pay attention to cultivar selection for *R. reniformis* management if varieties differ in susceptibility. The soybean cultivars used in this study varied by year due to seed availability, but there were not large fluctuations in *R. reniformis* abundances under soybean by year, indicating that cultivar was not a major factor. Rather, differences in *R. reniformis* abundances between soybean and cotton were more closely related to seasonal fluctuations in *R. reniformis* abundances under cotton. Soybean is a shorter season crop, and summer samples were done after all crops were harvested, so there was a longer fallow period after soybean than other crops, which may have also decreased *R. reniformis* abundances following that crop. In any case, this reflects a realistic rotation as fall planting of carinata is recommended – even after shorter season crops like soybean – to mitigate freeze risk (Seepaul et al., 2019).

Interactive effects of summer crops and winter rotations on *R. reniformis* management were expected based on crop host status, but crop effects generally did not vary based on prior crop. In year 2, carinata reduced *R. reniformis* abundances only in corn and not in other summer crops, but this was not observed in any other year. Overall, this suggests that carinata fits equally after host or non-host summer crops in terms of *R. reniformis* management.

Although this study provides insightful information about *R. reniformis* management using carinata as a rotational crop, further experiments should include carinata field rotations with carinata planted once in 3 yr. While a biennial frequency of carinata production was used for this study, production recommendations have since been updated to producing carinata one out of 3 yr to mitigate pathogen buildup (Seepaul et al., 2019). Testing of new carinata cultivars for their influence on *R. reniformis* management is also needed in the future. The carinata cultivar (Avanza 642) used in this study was the primary cultivar at the initiation of this study, but improved carinata cultivars have been released since that time and breeding work is ongoing.

## Conclusion

In conclusion, this study provides valuable insights that can guide crop selection decisions and expand the diversity of winter crops for nematode management in the Southeastern United States. The presence of *R. reniformis* below plow depth implies that deeper soil layers act as a source of this nematode. Limiting sampling to only the upper 30 cm may underestimate the total number of *R. reniformis* present throughout the soil profile. This underscores the necessity of comprehensive management strategies, such as crop rotation, beyond the soil depth that is commonly considered in nematode management (0–30 cm). Carinata does not provide consistent additional benefits or drawbacks compared to traditional fallow or oat in managing *R. reniformis*. Carinata fits equally well behind soybean, corn, peanut, or cotton summer crops as regards *R. reniformis* management.
